# Clinical Characteristics of Anti-3-Hydroxy-3-Methylglutaryl Coenzyme A Reductase Antibodies in Chinese Patients with Idiopathic Inflammatory Myopathies

**DOI:** 10.1371/journal.pone.0141616

**Published:** 2015-10-28

**Authors:** Yongpeng Ge, Xin Lu, Qinglin Peng, Xiaoming Shu, Guochun Wang

**Affiliations:** 1 Department of Rheumatology, China-Japan Friendship Hospital, 100029, Beijing, China; 2 Graduate School of Peking Union Medical College, 100730, Beijing, China; Nippon Medical School Graduate School of Medicine, JAPAN

## Abstract

**Objective:**

The objective of this study was to detect the prevalence of anti-3-hydroxyl-3- methylglutaryl coenzyme A reductase (anti-HMGCR) antibodies in Chinese patients with idiopathic inflammatory myopathies (IIMs), and to analyze the clinical features of the antibody-positive IIM patients.

**Methods:**

The presence of anti-HMGCR antibodies was detected in 405 patients with IIMs, 90 healthy controls, and 221 patients with other rheumatic diseases by using an ELISA kit. Clinical data from anti-HMGCR antibody-positive and -negative patients were compared. Long-term follow-up of the anti-HMGCR antibody-positive patients was conducted to evaluate the role of anti-HMGCR antibody in IIM disease prognosis.

**Results:**

Of the 405 IIM patients, 22 (5.4%) were found to carry the anti-HMGCR antibody. These IIM patients were predominantly female (73%), and only 3 anti-HMGCR antibody-positive patients with IIM were exposure to statins. Most patients experienced progressive onset, and presented with muscular weakness. Dysphagia was observed in half of the patients (*p* < 0.01), and 15% of these patients experienced the complication of interstitial lung disease (ILD) (*p* > 0.05). Mean creatine kinase (CK) levels were higher in antibody-positive patients than in antibody-negative patients (*p* < 0.05). Muscle biopsies were available from 12 anti-HMGCR antibody-positive patients, eight who experienced myofiber necrosis and showed very little or no evidence of inflammatory cell infiltrates in their muscle biopsies. Of these eleven patients who were followed-up 2.5- to 29-month, 73% experienced improvement after treatment. A cross-sectional study showed that anti-HMGCR antibody levels were significantly associated with CK levels (*r* = 0.486, *p* = 0.026) as well as with Myositis Disease Activity Assessment (MYOACT) scores (*r* = -0.67, *p* = 0.003) during the initial visit. However, changes in serum anti-HMGCR antibody levels did not correlate with changes in CK levels, Manual Muscle Testing 8 (MMT-8) scores or MYOACT scores in long-term follow-up.

**Conclusion:**

The major clinical features of anti-HMGCR antibody-positive Chinese IIM patients were muscle weakness and dysphagia, which were seen in patients with and without statin exposure. This subtype of patients were responsive to immunosuppressive treatment and received good prognoses after treatment, but serum levels of the anti-HMGCR antibody do not correlate with disease activity.

## Introduction

Idiopathic inflammatory myopathies (IIMs) are a group of clinically heterogeneous, autoimmune inflammatory muscle disorders characterized by muscle weakness and multisystem involvement. The main clinical subtypes of IIMs among Chinese populations are polymyositis (PM) and dermatomyositis (DM). The presence of myositis-specific autoantibodies (MSAs) is one hallmark of the disease [1]. The clinical importance of MSAs has gradually been recognized in recent years [2]. More and more studies show that different MSAs are characteristic of different clinical subtypes. For example, anti-melanoma differentiation-associated gene 5 (anti-MDA-5) antibodies are characteristic of a distinctive clinical subgroup with interstitial lung disease, and anti-transcription intermediary factor 1 (anti-TIF1) antibodies are characteristic of a subgroup of IIM patients who are at a high risk of developing cancer [3–4].

The anti-3-hydroxy-3-methylglutaryl coenzyme A reductase (anti-HMGCR) autoantibody was first reported by Christopher-Stine and colleagues as “anti-200/100.” Given the strong association of this novel autoantibody and associated necrotizing myopathy with statin use in 63% of the patients, an autoantigenic target in the cholesterol synthesis cascade was sought. Mammen and colleagues in the same group later identified the anti-200/100 autoantibody as anti-HMGCR. [5–6]. However, other studies have revealed that the majority of anti-HMGCR antibody-positive IIM patients from European cohort were not exposed to statins [7–9].

The prevalence of anti-HMGCR antibodies has never been investigated among Chinese IIM populations, and the clinical features of Chinese anti-HMGCR antibody-positive patients were previously unknown. To address these questions, we measured serum anti-HMGCR antibodies levels in 405 IIM patients and 311 controls, and compared the differences between the clinical features of the anti-HMGCR antibody-positive and -negative patients. The anti-HMGCR antibody-positive patients were also followed-up to analyze how this subgroup responded to therapy and whether levels of anti-HMGCR antibody could predict disease activity or disease prognosis.

## Materials and Methods

### Ethics statement

All samples were obtained for research purposes. In the retrospective study, patients’ consents were impossible or impractical to obtain. All patients data was anonymously used. This study was approved by the Research Review Committee (RRC) and the Ethical Review Committee (ERC) of the China-Japan Friendship Hospital.

### Patients

All IIM patients (n = 405) fulfilled the Bohan and Peter criteria [10–11] for PM and DM; 117 of these patients had PM and 288 had DM. Their sera were obtained between April 2009 and March 2015. The following data were obtained from their medical records: age, sex, past medical history, statin exposure, muscle strength, lung function, chest computed tomography (CT) images, CK levels, lactate dehydrogenase (LDH), hydroxybutyric acid dehydrogenase (HBDH), the presence of other MSAs, and levels of immunoglobulin (IgA, IgG, IgM), C-reactive protein (CRP), Complement 3 (C3) and Complement 4 (C4) as well as erythrocyte sedimentation rate (ESR). Muscle biopsy specimens of patients who carried anti-HMGCR antibodies were reviewed. Patients demonstrated muscle weakness, according to their Manual Muscle Testing 8 (MMT-8) scores, the maximum score being 80 [12]. The Modified Myositis Disease Activity Assessment Tool (MYOACT)–which assessed constitutional, cutaneous, skeletal, gastrointestinal, pulmonary, and cardiac conditions—was used to evaluate disease activity [13].

Patients who carried the anti-HMGCR antibody received follow-up, and their response to treatment was evaluated. Improvement was defined as an improvement of more than 20% on at least two of the study’s core measurement tools (e.g. MMT-8, MYOACT and serum CK levels), with the score on the third tool decreasing no more than 25%.

Our study controls included blood from 90 healthy donors and from 221 patients with other different inflammatory/autoimmune diseases, including 30 patients with primary Sjögren’s syndrome (pSS), 20 with ankylosing spondylitis (AS), 55 with systemic lupus erythematosus (SLE), 50 with rheumatoid arthritis (RA), 10 with systemic sclerosis (SSc), 30 with idiopathic hyper-creatine kinase-emia, 10 with muscular dystrophy, 6 with myasthenia gravis, 5 with motor neuron disease, and 5 with mitochondrial myopathy. Basic clinical data, including age and sex, were recorded for these controls samples. Sera from all groups were collected and stored at -80°.

### Quantifying anti-HMGCR antibody by ELISA

ELISA plates coated with recombinant HMGCR were incubated with diluted patient samples. Assay procedures followed the standard protocol of the QUANTA Lite assays (INOVA Diagnostics). Mean absorbance (OD) of the samples was plotted as a standard curve against their values in unites. A linear Cubic Spine (third order polynomial) fit or a fourth order polynomial fit was used to draw these curves. Unknown HMGCR concentrations were determined by finding unit values (U) on the Y axis and reading their corresponding absorbance values on the X axis. Negative values corresponded to Y-axis values 0–20 U. Positive values corresponded to Y-axis values greater than or equal to 20 U.

### Statistical analysis

All analyses were completed using SPSS 17.0, and *P* values less than 0.05 were considered significant. Correlation analyses were performed using the Spearman test. Quantitative variables are reported as means and compared using a nonparametric test. Categorical variables are reported as numbers and/or percentages and were compared using the Chi-square test or, when appropriate, Fisher’s exact test.

## Results

### 1. The frequency of anti-HMGCR antibodies in IIM patients

In total, 22 of the 405 patients (5.4%) with IIM carried anti-HMGCR antibodies (S1 Fig). Anti-HMGCR antibodies were not present in healthy controls or in disease control groups—i.e., patients with AS, RA, SLE, SSc, muscular dystrophy, myasthenia gravis, motor neuron disease, and mitochondrial myopathy. However, one pSS patient carried the anti-HMGCR antibody.

### 2. Clinical phenotype

#### 2.1 Clinical features of anti-HMGCR antibody-positive patients

The anti-HMGCR antibody was found in 2.8% (8/288) of DM patients and 12% (14/117) of PM patients (S1 Table). There were 16 (73%) females and six (27%) males among the anti-HMGCR antibody-positive patients. The average age and average disease duration of anti-HMGCR antibody-positive patients are similar to those of antibody-negative patients (both *p >* 0.05). In 3 of 20 patients (15%), the anti-HMGCR antibody was produced following exposure to statins (complete clinical data was not available for every patient). Two patients were administered by atorvastatin, rosuvastatin was administered to another one. Two patients had statin exposure in the five anti-HMGCR antibody-positive subjects over the age of 50 (40%), the prevalence of statin used in patients with anti-HMGCR antibody was higher than 28 of the 133 (21%) anti-HMGCR antibody-negative IIM patients over 50 years old, but the difference was not statistically significant (*p* > 0.05). No other relevant myotoxin exposures were identified upon review of the patient records.

Of these 20 patients, five (30%) experienced a subacute onset (< 12 months), 14 (70%) experienced a progressive onset (> 12 months). Of these same 20 patients, 15 (75%) patients presented with muscular weakness, and 14 (70%) patients suffered myalgia for the whole duration. Twelve patients experienced symmetric proximal muscle weakness at the time of the initial serum collection; the remaining three patients had a history of muscular weakness.

Half (50%) of these patients had dysphagia, and this incidence was higher than dysphagia incidence in the antibody-negative group (20%) (*p* < 0.01). Of the ten patients with dysphagia, 4 patients characterized by difficulty into solid food, 3 patients choking cough when drinking water and one of them had difficulty in speaking concurrently, and the other three patients required nasogastric tube and two of them had difficulty in speaking concurrently. Of the anti-HMGCR antibody-positive patients, five (25%) had arthralgia and three (15%) had ILD. But these numbers were not significantly different compared to those in the negative group (*p* > 0.05). Osteosarcoma was observed in one patient.

#### 2.2 Laboratory tests of anti-HMGCR antibody-positive patients

Eighteen of 21 (85.6%) anti-HMGCR antibody-positive patients had a history of elevated serum muscle enzyme levels, with mean CK levels of 2538.7 ± 3047.6 IU/L during their initial admission. Serum muscle enzyme levels—including CK, LDH and HBDH—in anti-HMGCR antibody-positive patients were significantly higher than those in antibody-negative patients (all *p* < 0.05). Although IgG, IgA and IgM levels in anti-HMGCR antibody-positive patients were all lower than those in the antibody-negative group, the difference was not statistically significant (all *p* > 0.05). Levels of CRP, C3 and C4 as well as ESR were also not significantly difference between the two groups. Anti-Jo-1 antibody was detected in three (14%) of the anti-HMGCR antibody-positive patients. Anti-SRP antibodies were not detected in all anti-HMGCR antibody-positive patients (S1 Table).

#### 2.3 Pathological features of muscles of antibody-positive patients

Muscle biopsies were available for 12 of the 22 anti-HMGCR antibody-positive patients. Eight patients (67%) suffered from significant myofiber necrosis with little or no inflammatory cell infiltration that could be diagnosed as immune-mediated necrotizing myopathy (IMNM) (S2 Fig), according to European Neuromuscular Centre criteria (ENMC) [14]. Two patients had necrotic myofibers along with obvious inflammation. Two subjects did not suffer from myofiber necrosis but did experience some inflammatory cell infiltration. Of the eight patients who experienced myofiber necrosis, four (50%) expressed MHC-I on the surface of regenerative or necrotic myofibers. Of the muscle biopsies from the three patients who had experienced statin exposure, two showed myofiber necrosis with little inflammation, and the remaining one only had inflammatory infiltrates.

### 3. Correlation analysis between disease activity and anti-HMGCR antibody levels

Anti-HMGCR antibody levels, serum CK levels, MMT-8 score, and MYOACT score were assessed in anti-HMGCR antibody-positive patients at the time of their initial evaluation (S3 Fig). Anti-HMGCR antibody levels had a strong correlation with MYOACT scores (*r* = -0.611, *p* = 0.02) but not with CK levels and MMT-8 scores (both *p* > 0.05) in patients without statin exposure. From an overall analysis of all anti-HMGCR antibody-positive patients, there was a statistically significant association between anti-HMGCR antibody levels and serum CK levels (*r* = 0.486, *p* = 0.026) (S3A Fig) and MYOACT scores (*r* = -0.67, *p* = 0.003) (S3B Fig), but there was no significant correlation between anti-HMGCR antibody levels and muscle strength (MMT-8 score) in these patients (*r* = -0.16, *p* = 0.545).

### 4. Follow-up study of anti-HMGCR antibody-positive patients

Among anti-HMGCR positive patients, 11 patients were followed longitudinally, including two patients with previous statin exposure. The median follow-up time was 9 months, with a range of 2.5 months to 2 years. Anti-HMGCR antibody levels, serum CK levels, MMT-8 scores, and MYOACT scores were recorded at each visit for monitoring (S4 Fig).

Of these 11 patients, eight (73%) responded well to glucocorticoids and/or other immunosuppressive agents; either their MMT-8 scores (S4A Fig) and MYOACT scores improved (S4B Fig) or their CK levels declined (S4C Fig). The average glucocorticoid dosage was reduced from 63.1 mg/day to 20.5 mg/day for these patients. Furthermore, three patients—including two with prior statin exposure—had near-complete remission after treatment; they recovered normal muscle strength and normal CK levels, and suffered very little extramuscular disease activity. Three of these 11 patients (27%) did not experience improvement according to our criteria for improvement. Patients who deteriorated were younger on average than those who experienced improvement (mean ages: 27.7 years VS 41.9 years, respectively).

The longitudinal data points representing anti-HMGCR antibody levels in seven patients (S4D Fig). Despite the decline in anti-HMGCR antibody levels over time in some patients after treatment, the antibody levels had not normalized in six (85.7%) of the seven patients by the last study visit, despite some patients regaining normal muscle strength. The antibody levels of only one patient—whose anti-HMGCR antibody titer was 22 U—decreased to fall within the normal range during the observation period.

After treatment, antibody levels changed, but the change in serum antibodies levels did not correlate with changes in CK levels, MMT-8 scores and MYOACT scores in the seven patients (all *p* > 0.05).

## Discussion

This is the first study to describe the prevalence of anti-HMGCR antibodies in a large sample of Chinese IIM patients. We found that anti-HMGCR antibody-positive patients exhibited a slow onset of disease and presented with muscle weakness and dysphagia, which were seen in patients with and without statin exposure. Most patients experienced myofiber necrosis with little or no inflammatory cell infiltration in their muscle biopsies. Most of the patients responded well to immunosuppressive treatment and achieved a good prognosis, but serum levels of the anti-HMGCR antibody did not correlate with disease activity.

The present study shows that the anti-HMGCR antibody is present in 5.4% of patients with IIM, similar to findings from two other large sample studies [6, 15]. But this antibody is also present in patients with other autoimmune diseases. We detected the antibody in one pSS patient, but that titer was lower. Similar results were also reported by Musset L et al [9]. In our study, the pSS patients with anti-HMGCR antibody did not have muscular weakness, shin rash, elevated CK level and other MSAs from 2009 to July of this year.

In our study, the mean age of anti-HMGCR antibody-positive IIM patients was similar to the age of antibody-negative patients. Mammen et al showed that the mean age of anti-HMGCR antibody-positive patients without statin exposure was lower than that of statin-exposed antibody-positive patients [6]. Because of the small number of patients who had prior statin exposure in this study, it was difficult to make comparisons between antibody-positive patients with statin exposure and those who had never been exposed. Only 15% of anti-HMGCR antibody-positive patients in the current study had previous statin exposure. This percentage is lower than the 44% and 72.7% reported in studies by Allenbach et al and Werner JL et al, respectively [8, 15]. Since hyperlipidemia increases with age, the prevalence of statin use increases with age. So the Hopkins group compared statin exposure prevalence of anti-HMGCR antibody-positive patients with age-matched antibody-negative patients. They found that the prevalence of statin use in anti-HMGCR patients was significantly higher than the rates of statin use in age over 50 years-old groups of PM, DM, and IBM patients [5–6]. But we did not find the association in older patients, may be due to the small number of antibody-positive patients in our study. This should be confirmed by large-sample studies. However, the reason for this lower percentage is not that statins are prescribed less often in China, In fact, tens of millions of Chinese patients suffer from hyperlipidemia, and the majority of them are given statins to lower cholesterol. The lower percentage in our study may suggest that different genetic backgrounds may trigger the occurrence of anti-HMGCR antibodies in different populations.

Regarding the type of disease onset, most of the patients in our study experienced a muscular weakness whose slow progression ranged from 12 months to a few years. In contrast, subacute onset was the main onset type in European patients [8] and in Japanese patients [16]. This difference may suggest that the onset of anti-HMGCR antibody-associated IIM varies between different races and ethnicities.

The main clinical features of muscular weakness and myalgia were observed both in our cohort and in other study cohorts [6, 8]. However, another prominent clinical feature of antibody-positive patients in our cohort was dysphagia. Christopher-Stine et al also noted dysphagia in 63% of their patients with the anti-HMGCR antibody [5]. But, Allenbach Y et al reported that 26.7% of their European patients presented with dysphagia [8], and Watanabe Y et al observed no swallowing disorders in their Japanese patients [16]. This difference may show that heterogeneity of dysphagia in different group.

We observed that three patients carried both anti-HMGCR and anti-Jo-1 antibodies. This suggests that the anti-HMGCR antibody could coincide with other MSAs. Mammen et al also reported that one patient with anti-HMGCR antibody had Jo-1 antibodies with ILD and another patient had scleroderma with positive anti-Pm/Scl titers and ILD [6]. Interestingly, two of the three patients with the anti-Jo-1 antibody did not have ILD in our study. Because anti-Jo-1 antibodies were closely associated with ILD, so we couldn’t rule out the possibility of anti-Jo-1 antibody false positivity in the other two patients.

We reviewed the muscle biopsies of anti-HMGCR antibody-positive patients, most of them associated with the IMNM We also found necrotic myofibers along with mild inflammation or only inflammation in muscle biopsies of other patients,. This finding shows that muscle histopathology varies among anti-HMGCR antibody-positive patients. This variation was also observed by Allenbach Y et al [8]. MHC-I expression was detected in half of the antibody-positive patients, consistent with findings from the Johns Hopkins cohort, and four of the eight available biopsy specimens included sarcolemmal MHC-I staining of myofibers [5].

Werner JL et al demonstrated that anti-HMGCR antibody levels had a statistically significant association with CK levels and muscle strength when statin-exposed and statin-unexposed patients were examined together during their initial visit. When analyzed separately, these associations were confirmed in the antibody-positive patients who had prior statin exposure, but not in antibody-positive patients with no prior statins exposure [15]. Our cross-sectional study shows that anti-HMGCR antibody levels do not correlate with muscle strength but are significantly associated with serum CK levels. In patients with no statin exposure, anti-HMGCR antibody levels only correlated with MYOACT scores. Because only three anti-HMGCR patients were exposed to statins in our study, we were unable to define the relationship between antibody levels and CK levels or muscle strength in statin-exposed patients.

Furthermore, we studied some patients longitudinally to see how antibody-positive patients responded to treatment. In the present study, we found that most anti-HMGCR antibody-positive patients respond well to treatment, especially patients with previous statin exposure. This findings is similar to that in the Watanabe Y et al study, in which seven patients who were followed for two years all responded well to immunotherapy [16]. Additionally, this study shows that patients whose conditions did not improve after treatment were younger than those who improved after treatment. This phenomenon may show that anti-HMGCR antibody-positive patients with early onset are refractory to treatment.

In order to understand whether the anti-HMGCR antibody reflects disease activity, we detected serial changes in the antibody levels of seven patients. We found that the antibody levels did not decrease significantly in patients who got improvement. Some patients had elevated antibody levels even after complete remission. In our cohort, changes in antibody levels did not significantly correlate with lower CK levels or improved muscle strength in the follow-up period. Therefore, anti-HMGCR antibody levels may not be a useful indicator of disease activity. This finding differed from those from the Australian cohort; they reported that immunosuppressive therapy caused antibody and CK levels to decline, with a corresponding improvement in muscle strength during long-term follow-up [17].

We recognize that this study has several limitations. First, the study involves a relatively small number of patients for follow-up, and some of whom were only followed for a short time. Second, because this was a retrospective study, treatment plans and the time intervals between each visit were not standardized. Furthermore, some patients had already received immunotherapy before admission to our hospital, so our initial visit data do not reflect these patients’ conditions prior to treatment. Lastly, complete clinical data was not available for every patient at every visit.

In conclusion, our study shows that the major clinical features of anti-HMGCR antibody-positive Chinese IIM patients are muscle weakness and dysphagia, which were seen in patients with and without statin exposure. Our study also shows that these patients respond well to immunosuppressive treatment and achieve good post-treatment prognoses. All of these findings suggest a phenotypic difference between anti-HMGCR antibody-positive patients and antibody-negative patients. Anti-HMGCR antibody-associated IIM may be a distinct clinical phenotype. However, additional studies involving larger numbers of patients over longer time periods are required to confirm this conclusion.

## Supporting Information

S1 Fig Anti-HMGCR antibody presence in each group. (DOCX)

S2 Fig Histologic and immunohistologic analysis of muscle biopsies. (DOCX)

S3 Fig Anti-HMGCR antibody levels correlate with CK levels and MYOACT scores. (DOCX)

S4 Fig Follow-up findings from anti-HMGCR antibody-positive patients. (DOCX)

S1 Table Characteristics of anti-HMGCR antibody-positive patients. (DOCX)
