# Supplementary figures and images for: Clinical Characteristics of Anti-3-Hydroxy-3-Methylglutaryl Coenzyme A Reductase Antibodies in Chinese Patients with Idiopathic Inflammatory Myopathies
Source: PLoS One. 2015 Oct 28;10(10):e0141616. doi: 10.1371/journal.pone.0141616 (PMC4624805; doi:10.1371/journal.pone.0141616)

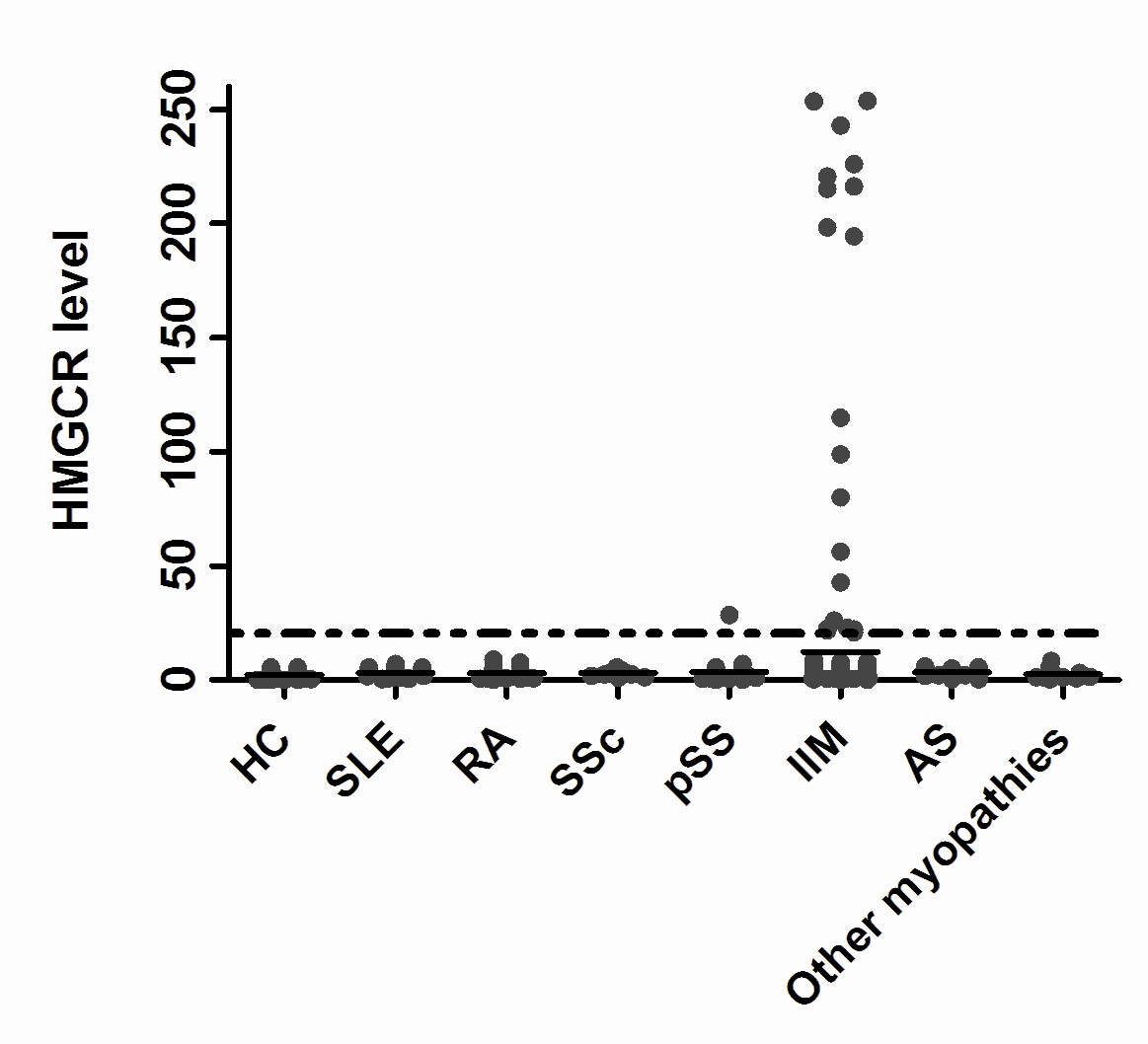


**Fig 1. Anti-HMGCR antibody presence in each group**

Supplement: S1 Fig — (DOCX) [file pone.0141616.s001.docx]
