# Supplementary material for: Clinical Characteristics of Anti-3-Hydroxy-3-Methylglutaryl Coenzyme A Reductase Antibodies in Chinese Patients with Idiopathic Inflammatory Myopathies
Source: PLoS One. 2015 Oct 28;10(10):e0141616. doi: 10.1371/journal.pone.0141616 (PMC4624805; doi:10.1371/journal.pone.0141616)
Supplement: S2 Fig — (DOCX) [file pone.0141616.s002.docx]

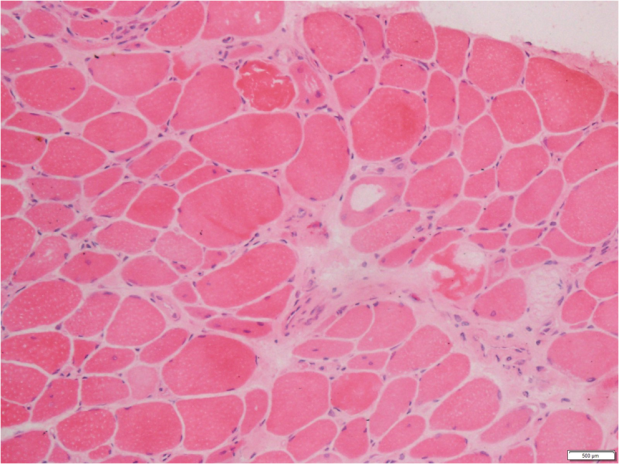

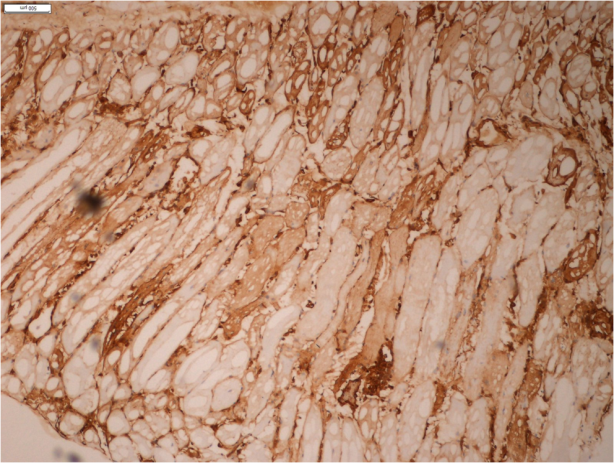


A B

**Fig 2. Histologic and immunohistologic analysis of muscle biopsies show necrotic and degenerated fibers from the biceps of an anti-HMGCR antibody-positive patient**

**(A) stain with HE (B) stain with anti-MHC-I antibody**
