# Supplementary material for: Clinical Characteristics of Anti-3-Hydroxy-3-Methylglutaryl Coenzyme A Reductase Antibodies in Chinese Patients with Idiopathic Inflammatory Myopathies
Source: PLoS One. 2015 Oct 28;10(10):e0141616. doi: 10.1371/journal.pone.0141616 (PMC4624805; doi:10.1371/journal.pone.0141616)
Supplement: S3 Fig — (DOCX) [file pone.0141616.s003.docx]

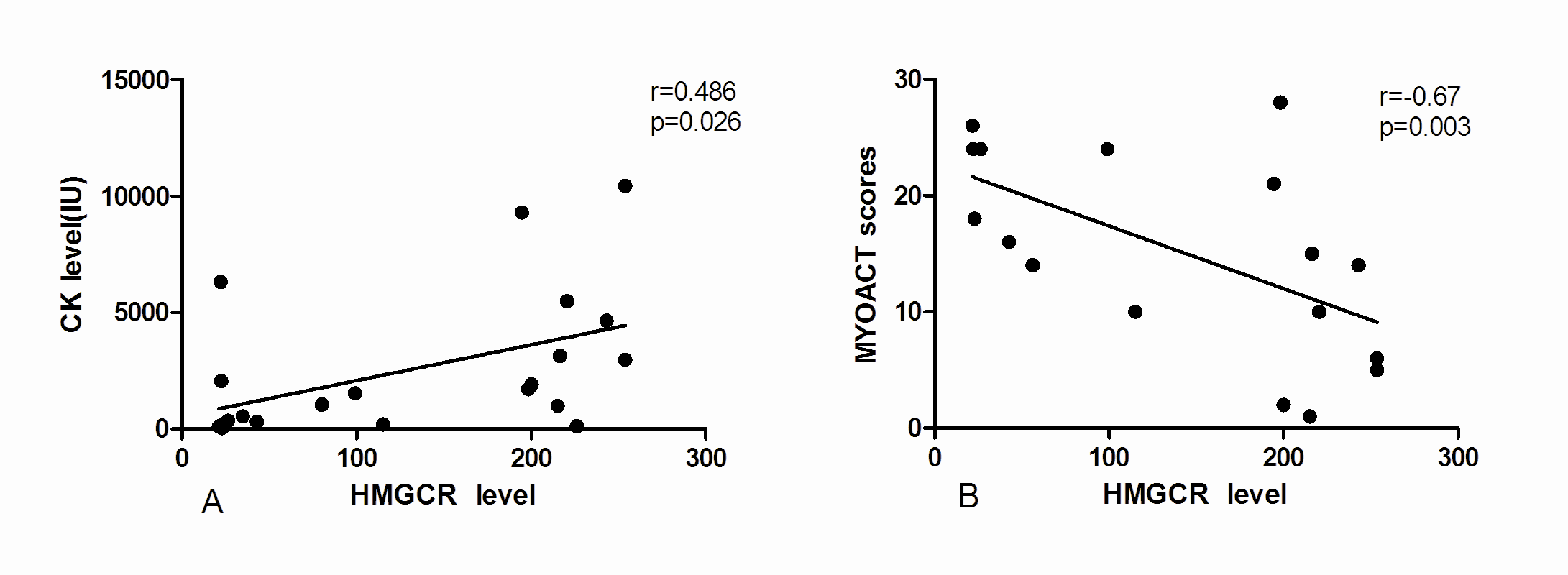


**Fig 3. Anti-HMGCR antibody levels correlate with CK levels and MYOACT scores: Significant correlations were observed between anti-HMGCR antibody levels and serum CK levels (A) and MYOACT scores (B).**
