# Supplementary material for: Clinical Characteristics of Anti-3-Hydroxy-3-Methylglutaryl Coenzyme A Reductase Antibodies in Chinese Patients with Idiopathic Inflammatory Myopathies
Source: PLoS One. 2015 Oct 28;10(10):e0141616. doi: 10.1371/journal.pone.0141616 (PMC4624805; doi:10.1371/journal.pone.0141616)
Supplement: S4 Fig — (DOCX) [file pone.0141616.s004.docx]

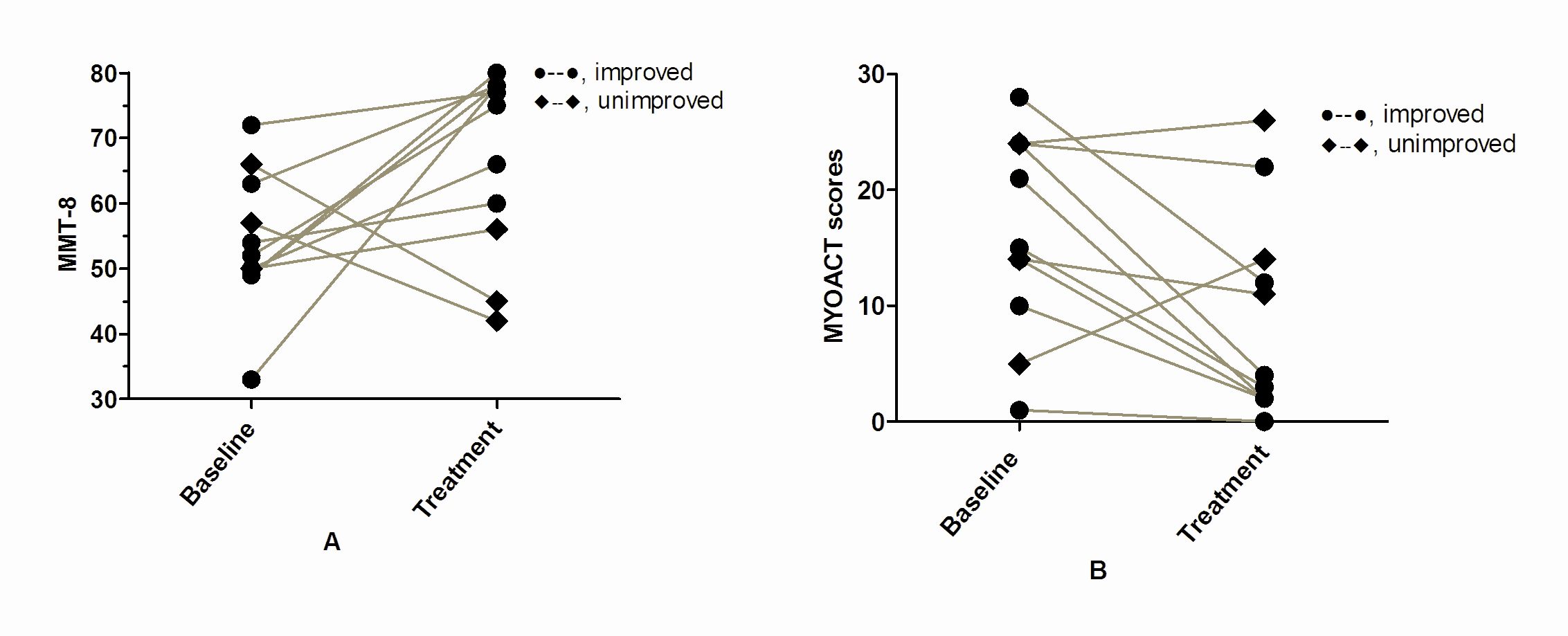


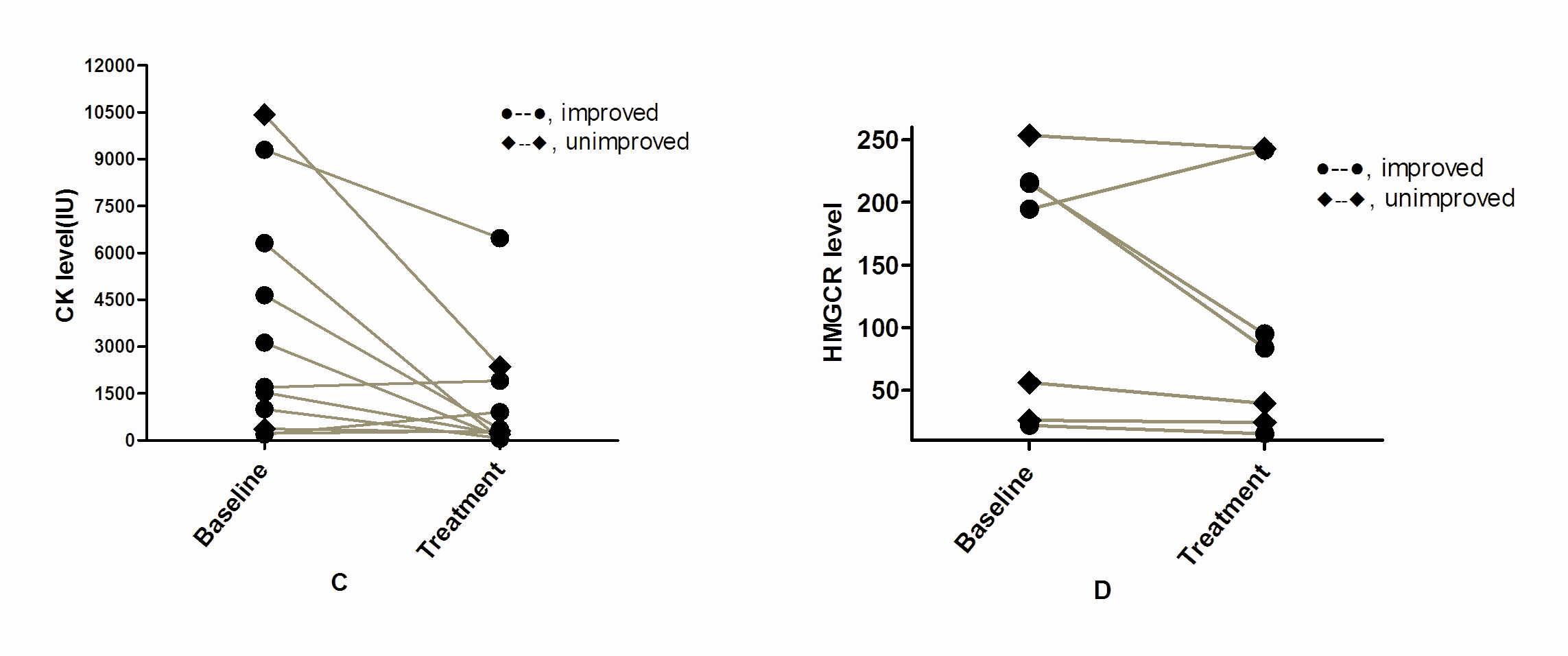


**Fig 4. Follow-up findings from anti-HMGCR antibody-positive patients: MMT-8 scores (A), MYOACT scores (B), CK levels (C), and anti-HMGCR antibody levels (D) at baseline and after treatment.**
