# Supplementary material for: Clinical Characteristics of Anti-3-Hydroxy-3-Methylglutaryl Coenzyme A Reductase Antibodies in Chinese Patients with Idiopathic Inflammatory Myopathies
Source: PLoS One. 2015 Oct 28;10(10):e0141616. doi: 10.1371/journal.pone.0141616 (PMC4624805; doi:10.1371/journal.pone.0141616)
Supplement: S1 Table — (DOCX) [file pone.0141616.s005.docx]

**Table 1. Characteristics of anti-HMGCR antibody-positive patients**

| Characteristic | anti-HMGCR antibody-positive patients | | | anti-HMGCR antibody-negative patients |
| --- | --- | --- | --- | --- |
| PM | | 14 | 117 | |
| DM | | 8 | 288 | |
| Female : Male | | 16:6 | 251:132 | |
| Age (years) | | 41.1 ±14.4 | 45.2 ±16.1 | |
| Disease duration (months) | | 31.2 ± 59.2 | 28.5 ± 48.1 | |
| Disease onset | |  |  | |
| Subacute onset (<12 months) | | 30% (6/20) | 52% (169/325) | |
| Progressive onset (>12 months) | | 70% (14/20) | 48% (156/325) | |
| Dysphagia | | 50% (10/20) | 20% (70/345)* | |
| Arthralgia | | 25% (5/20) | 31% (102/330) | |
| ILD | | 15% (3/20) | 31% (95/305) | |
| Anti-Jo-1 | | 14% (3/22) | 15% (45/294) | |
| Anti-SRP | | 0% (0/22) | 7% (9/125) | |
| CK | | 2538.7 ± 3047.6 | 1067.7 ± 2239.6* | |
| LDH | | 618.7 ± 299.1 | 401.5 ±358.2* | |
| HBDH | | 395.0 ± 213.6 | 274.4 ± 260* | |
| IgG | | 1002.2 ± 485.7 | 1245.2 ± 597.6 | |
| IgM | | 125.7 ±82 | 168.9 ± 576.8 | |
| C3 | | 92.0 ± 16.5 | 90.2 ± 22.2 | |
| C4 | | 19.7 ± 3.4 | 20.2 ± 6.8 | |
| CRP | | 2.1 ± 6.0 | 2.1 ± 7.1 | |
| ESR | | 17.2 ± 13.9 | 27.3 ± 27.1 | |

ILD, interstitial lung disease; CK, creatine kinase; LDH, lactate dehydrogenase; HBDH, hydroxybutyric acid dehydrogenase; C3, complement 3; C4, complement 4; Ig, immunoglobulin; CRP, C-reactive protein; ESR, erythrocyte sedimentation rate; *, *p* < 0.05; NA, not available
